# A Rare Case of Odontogenic Chronic Suppurative Otitis Media

**DOI:** 10.7759/cureus.4284

**Published:** 2019-03-20

**Authors:** Derrek A Schartz, Marc A Polacco, Eric P Holmgren, Ryan R McCool

**Affiliations:** 1 Otolaryngology, Dartmouth Geisel School of Medicine, Lebanon, USA; 2 Otolaryngology, Dartmouth-Hitchcock Medical Center, Lebanon, USA; 3 Oral and Maxillofacial Surgery, Dartmouth-Hitchcock Medical Center, Lebanon, USA

**Keywords:** chronic otitis media, otorrhea, dental abscess, maxillary sinusitis

## Abstract

Common causes of chronic suppurative otitis media (CSOM) include persistence of acute otitis media, cholesteatoma, and eustachian tube dysfunction. We describe a patient who presented with CSOM of several years duration refractory to medical management. Ultimately, a dental abscess was found on computed tomography (CT) to be the source of concurrent ipsilateral maxillary sinusitis and mastoiditis. Extraction of the molar abscess resulted in complete resolution of her CSOM and need to be on antibiotics. To our knowledge, this is the first report of an odontogenic cause of chronic suppurative otitis media.

## Introduction

Patients with chronic suppurative otitis media (CSOM) suffer from perforation of the tympanic membrane (TM) accompanied by intermittent or constant purulent drainage lasting greater than six weeks [1]. The average global incidence of CSOM is 4.8 new cases a year per 1,000 people of all ages [2]. First-line therapy of ototopical quinolone combined with aural toilet is usually successful and oral antibiotics may be utilized if initial therapy fails [3,4]. Recalcitrant CSOM, despite appropriate medical treatment, may imply sequestered foci of bacterial infection or biofilm within the mastoid or middle ear, cholesteatoma, or repeated contamination of the middle ear through a tympanic membrane perforation or via the eustachian tube [1,4,5]. In the latter scenario, potential sources of bacterial contamination include infections of the sinuses and adenoids [6,7]. While others have documented cases of sinus disease resulting in otitis as well as dental disease leading to sinusitis, we report the first case of odontogenic CSOM.

## Case presentation

A generally healthy 88-year-old female with a history of chronic sinusitis and dental decay developed a perforation in her left tympanic membrane with purulent drainage. A local otolaryngologist treated her with ototopical antibiotics and the perforation and drainage resolved. However, shortly after stopping antibiotics, the problem returned, treatment was repeated, problem resolved, but resolution was temporary. After repeating this cycle for one year, the patient presented to our clinic in October 2016. Her presenting symptoms included purulent otorrhea and mixed hearing loss without otalgia or vertigo. Otomicroscopy revealed a normal right tympanic membrane and purulent fluid filling the left external auditory meatus. Evacuation of the purulence revealed a 1 mm perforation in the anterior-inferior TM with surrounding erythema and purulence in the middle ear. Nasopharyngoscopy revealed normal nasal, nasopharyngeal, and eustachian tube anatomy. Her left ear was cleaned, and she was prescribed otic ciprofloxacin-dexamethasone drops as well as oral trimethoprim-sulfamethoxazole (TMP-SMX) due to history of amoxicillin-clavulanate allergy. Her otorrhea ceased after 48 hours and her antibiotic course continued for three additional weeks, during which time she had no drainage and examination showed closure of her perforation. However, one week after finishing the antibiotic course, her otorrhea recurred. Cultures were obtained and returned positive for TMP-SMX sensitive Staphylococcus aureus. A three-month course of TMP-SMX was initiated in conjunction with topical antibiotics, resulting in complete resolution of drainage. One month after finishing the second course, her symptoms returned; she was restarted on oral TMP-SMX and drainage once again subsided.

Computed tomography (CT) imaging was obtained and demonstrated left mastoid opacification and dehiscence of the bony septation between the mastoid opacification and the sigmoid sinus. At this time, the etiology of her symptoms was suspected to be secondary to chronic mastoiditis, and since durable resolution of otorrhea with extended antibiotic therapy could not be achieved, tympanoplasty with mastoidectomy was recommended. However, the patient was strongly averse to surgical treatment, and preferred to take TMP-SMX indefinitely rather than have the operation. In September 2017, a magnetic resonance imaging (MRI) was ordered to ensure that the bony destructive changes adjacent to the sigmoid sinus on her CT did not represent the effects of a cholesteatoma. The MRI revealed “a focus of restricted diffusion with imaging suggesting a small cholesteatoma,” and surgery was recommended. The patient again opted to continue treatment with chronic oral TMP-SMX rather than undergo surgery. In December 2017, her CSOM broke through and became symptomatic while on chronic oral TMP-SMX. Topical ciprofloxacin-dexamethasone was added and her suppuration resolved within a week.

**Figure 1:**
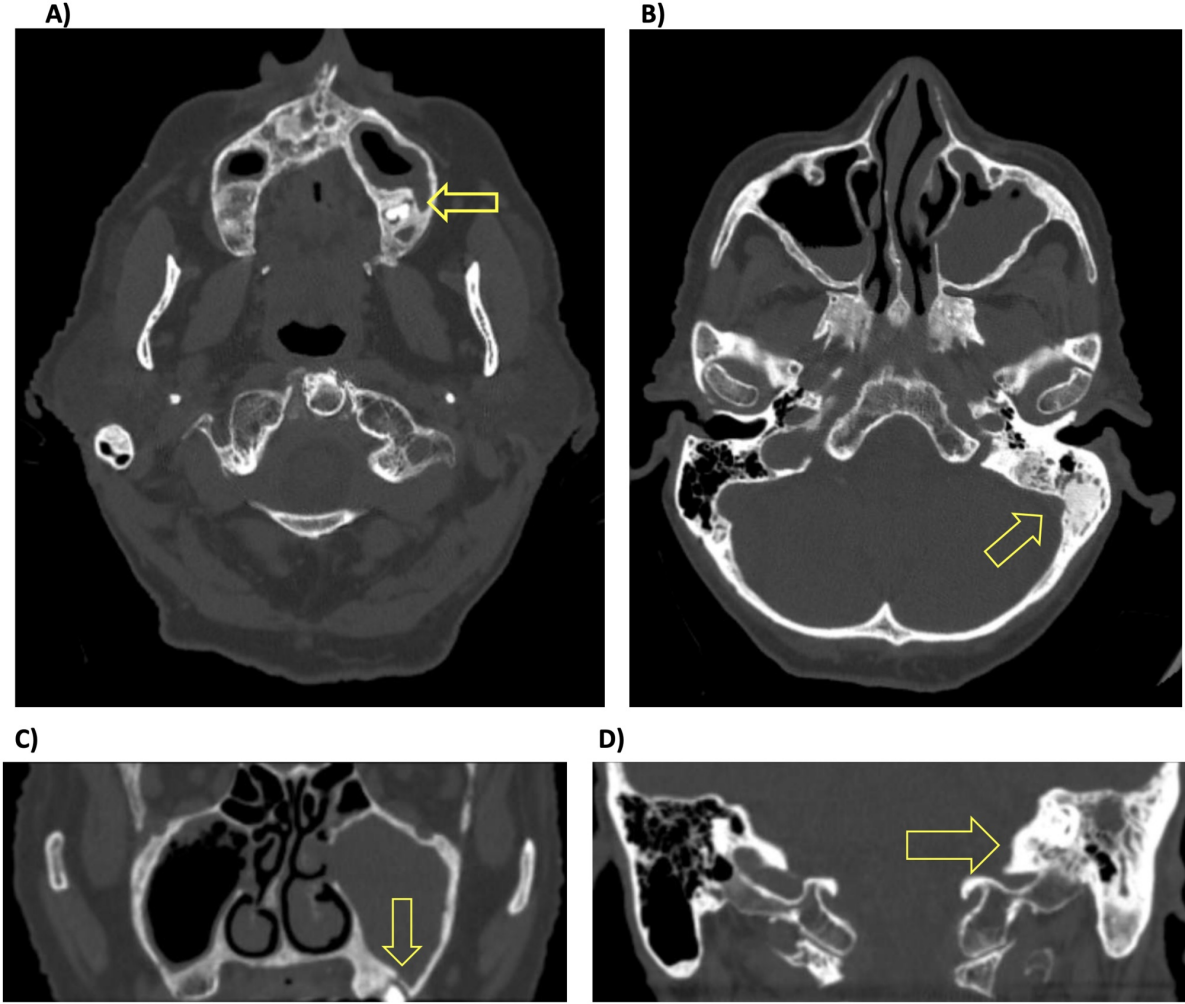
Computed tomography image of communication between periapical abscess and maxillary sinus, with ipsilateral maxillary sinusitis and mastoiditis. (A) Axillary view showing communication between molar abscess and maxillary sinus. (B) Axillary view demonstrating ipsilateral maxillary sinusitis and mastoiditis. (C) Coronal view of communication. (D) Coronal view of ipsilateral mastoiditis.

In January 2018, a new temporal bone CT showed improved pneumatization of the left mastoid and middle ear without any opacification concerning for cholesteatoma. At this point, we began looking for another source of recurrent contamination of the middle ear and mastoid air cells that could explain her repeated relapses after discontinuation of systemic and topical antibiotics. All previous CT and MRI images had demonstrated ipsilateral maxillary sinus inflammation and/or air-fluid levels. Closer review of her CT imaging revealed a periapical lucency of tooth #15 with communication with the left maxillary sinus (Figure 1).

**Figure 2:**
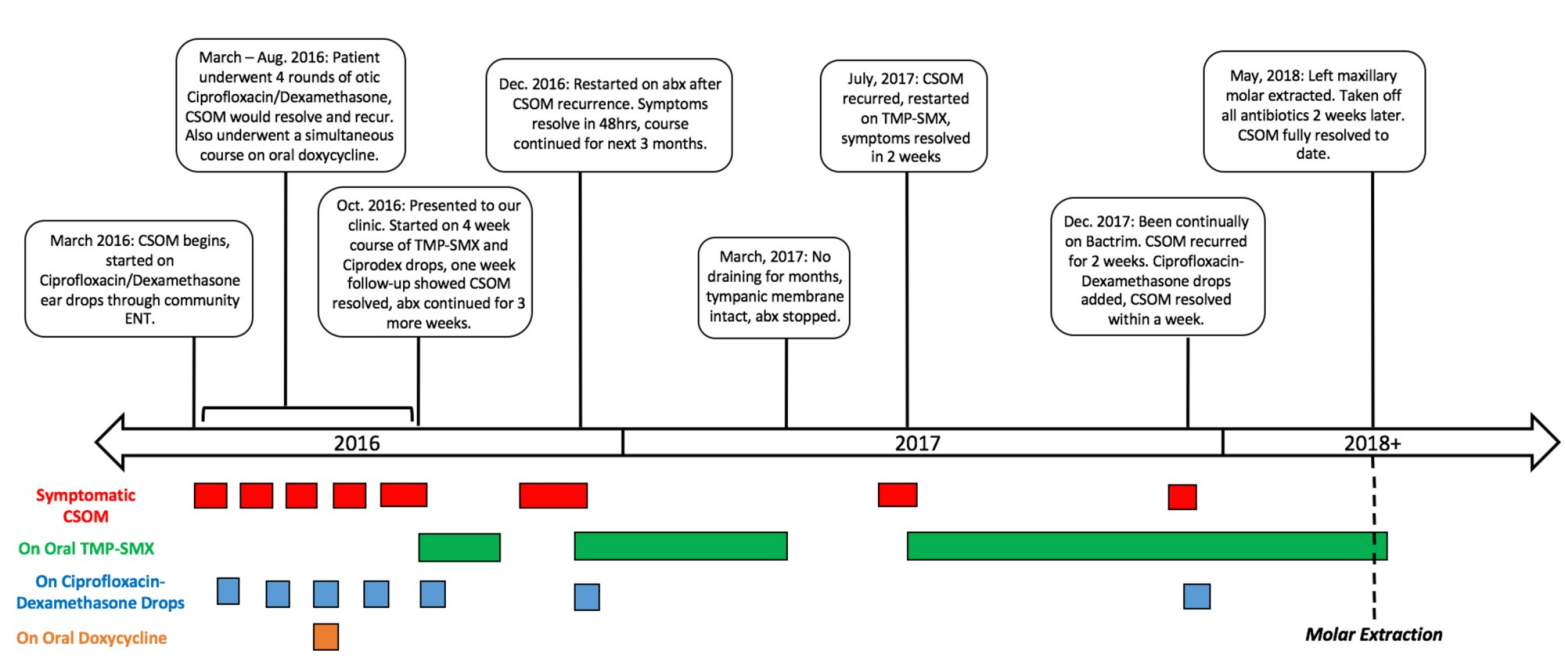
Summary of the timeline and relationship between symptomatic chronic suppurative otitis media and antibiotic treatment. CSOM: Chronic suppurative otitis media; ENT: Ear nose and throat (surgeon); TMP-SMX: Trimethoprim-sulfamethoxazole; abx: Antibiotics.

Oral examination then revealed the tooth to be loose to gentle palpation without tenderness or purulence, a finding consistent with periapical abscesses that are freely draining into the maxillary sinus [8]. This provided evidence that the left maxillary molar was functioning as a nidus for recurrent infection of the left maxillary sinus, resulting in spread of infection through the eustachian tube into the middle ear. As a result, molar extraction was recommended and completed, and all antibiotic therapy was discontinued two weeks later. At a six-month follow-up the patient remained off of antibiotics with complete resolution of otorrhea and significant improvement in hearing. At the time of submission - nine months after molar extraction - the patient remained symptom free. The patient’s disease course is summarized and depicted in Figure 2.

## Discussion

Here we present a patient with chronic suppurative otitis media which responded completely to topical and systemic antibiotics but recurred promptly with treatment cessation. Investigation for distant sources of middle ear contamination revealed odontogenic sinusitis. Ultimately, dental extraction produced durable resolution of CSOM after stopping all antibiotics.

Common causes of CSOM include persistence of acute otitis media, eustachian tube dysfunction, and cholesteatoma within the middle ear [1]. Chronic perforations of the tympanic membrane facilitate contamination of the middle ear from the ear canal or through the eustachian tube due to loss of the protective “middle ear cushion” [9]. In these cases, tympanoplasty is often the best treatment for the draining ear [10]. This case is different because the perforation healed spontaneously between episodes and only opened again to allow drainage of the recurring middle ear infection. For this reason, we theorized that this process was perpetuated by a sequestered infection in the mastoid or middle ear, cholesteatoma, or recurrent ascending contamination through the eustachian tube. Imaging initially suggested cholesteatoma, and we recommended mastoidectomy on several occasions after failed medical therapy. This patient’s decision to avoid mastoidectomy was ultimately validated, and this resulted in a lengthy timeline of relapsing CSOM which only durably resolved after management of the dental nidus for her ipsilateral maxillary sinus infection.

One important limitation to our conclusion is the lack of sinus or dental culture to compare to the ear culture. Her ear culture grew Staphylococcus aureus, which is a common cause of chronic odontogenic sinusitis [11, 12], and it is responsible for 21% of aerobic cases of concurrent chronic otitis media and chronic sinusitis [13]. Altogether, the evidence indicates that the patient’s periapical dental abscess was responsible for her maxillary sinusitis, and in turn, her CSOM.

This cause of CSOM has not been well described in the literature. A weak association between ear infections and dental caries has been reported in the past [14], and this case provides further evidence to support this. This case also demonstrates the importance of considering extra-otologic causes in similar non-resolving cases when the disease course is abnormal or the exact etiology is unclear. Imaging was also critical to diagnosis. When evaluating maxillary sinusitis of suspected odontogenic causes, CT is considered the gold standard [15]. This case also presents an excellent reminder to consider the whole patient and to maintain a broad differential when approaching complex and recalcitrant conditions.

## Conclusions

This is the first report documenting an odontogenic etiology of chronic suppurative otitis media. This report also provides further evidence for the potential link between ear and dental infections. While this has been investigated before, further studies are required to fully elucidate this potential connection. Additionally, this case serves as a good example to the sub-specialist about the importance of avoiding a tunnel-vision differential when examining patients.
